# Ethical considerations of universal vaccination against human papilloma virus

**DOI:** 10.1186/1472-6939-15-29

**Published:** 2014-04-07

**Authors:** Pedro Navarro-Illana, Justo Aznar, Javier Díez-Domingo

**Affiliations:** 1 Facultad de Enfermería, Universidad Católica de Valencia “San Vicente Mártir”, Valencia, Spain; 2 Instituto de Ciencias de la Vida, Universidad Católica de Valencia “San Vicente Martir”, Valencia, Spain; 3 Vaccine Research Department, FISABIO-Public Health, Valencia, Spain

**Keywords:** Human papilloma virus, HPV, Universal vaccination, Ethical assessment

## Abstract

**Background:**

From an epidemiological perspective, the practice of universal vaccination of girls and young women in order to prevent human papilloma virus (HPV) infection and potential development of cervical cancer is widely accepted even though it may lead to the neglect of other preventive strategies against cervical cancer.

**Discussion:**

It is argued that removing the deterrent effect – the fear of developing cancer – could encourage teenage sex. This paper reflects on the ethical legitimacy of the universal vaccination of girls and young women against HPV infection, especially regarding safety issues, the need to vaccinate people who have opted to abstain from sex, the presumption of early onset of sexual relations, the commercial interests of the companies that manufacture the vaccine, and the recommendation of universal vaccination in males.

**Summary:**

Based on the aforementioned information, we believe that the universal vaccination against HPV in young women is acceptable from an ethical point of view, given the medical advantages it presents.

## Background

Human papilloma virus (HPV) is routinely found in humans. There are more than 100 different types of HPV, but not all are health threatening. Some cause common condylomas and others plantar warts, but there is a group of HPVs that mainly infects the mucous epithelium of the anogenital tract and has an oncogenic nature [1].

Around 40 types of HPV are transmitted primarily through sex, causing a mucous infection, especially in the anogenital region [1]. Condoms do not appear to be an effective solution to prevent their transmission [2-8].

In the reproductive stage of its life cycle, HPV multiplies inside the infected cell, causing cell alterations that lead to morphological changes in the epithelial tissue and other types of lesions [9].

Genital infections due to HPV are very common. Most occur in young women although 70% disappear spontaneously within one year and 90% within two years [10]. When this type of infection does not remit spontaneously and persists over time, it can become a precursor of high-grade lesions [9,10] which can subsequently develop into cancerous lesions; it has even been stated that HPV infection is necessary for the development of cervical cancer in around 98% of cases [11,12].

Of the more than 100 different variants of HPV, [13] types 16 and 18 cause more than 70% of cervical cancers in Europe [14]. Furthermore, cervical cancer is the second most common cancer in women worldwide, after breast cancer [15,16]. In addition, HPV infection is also responsible for other types of cancer in women, such as vaginal and vulvar cancer, and also for certain cancers in males, such as oropharyngeal, penile, and anal canal cancer; [3,11]. HPV infection is present in more than 85% of cases of the latter [17].

### Incidence

Worldwide, HPV infection is responsible for more than half a million cases of cancer and more than 250,000 deaths every year [15,16]. The highest incidence occurs in developing countries, mainly due to a lack of resources in secondary prevention and treatment of the disease, and poor sex education of the population [15,16,18,19]. HPV infection is not yet a major problem in Spain, which to date has one of the lowest cervical cancer rates in the world [20,21] with mortality rates below 2.2/100,000 women/year, and fewer than 1000 deaths annually [22].

### Prevention

The most effective measure for preventing cervical cancer in the last century was the introduction of cervical screening programmes, especially the Papanicolaou test, for the early detection of precancerous lesions in women that were already infected (secondary prevention). This test has high specificity, but low sensitivity and so erroneous results are not uncommon [23]. Furthermore, not all women worldwide have had access to the test – especially in developing countries, but also in developed countries – so it has not been effective at a global level. There are currently other secondary prevention strategies, such as the HPV determination test [24] which is a more sensitive technique than the Papanicolaou test and causes fewer incorrect results. However, its generalised implementation in industrialised countries is in its early stages.

Even though an intervention on the modifiable risk factors (i.e. oral contraception, [25] smoking, [26] age of first sexual intercourse, promiscuity, [27] or other infections, [28] etc.) would be the most effective action to prevent infection, it has not had a major impact on the elevated incidence of infection and cervical cancer so far. For this reason, it has been necessary to develop alternatives to complement screening programmes [29,30]. In this respect, universal HPV vaccination has been proposed following the success of vaccination for the prevention of other types of infections [31].

### HPV vaccination

In 2006, the WHO recognised the high efficacy (99.8%) of the HPV vaccine in preventing moderate and severe precancerous cervical lesions associated with HPV types 16 and 18 in women not previously infected by these types [32-35] as well as the safety of the anti-HPV tetravalent vaccine [36]. Accordingly, the Food and Drug Administration (FDA) and European Medicines Agency (EMEA) approved the administration of the HPV vaccine in humans at the end of the same year, authorising its administration to girls from the age of 9 years. Its use was recommended at this early age (between 9 and 14 years) due to its greater immunogenicity with respect to later ages, and to obtain protection before the first sexual relations [37-40].

### Some problems that may occur due to the universal use of the HPV vaccine

For the reasons previously stated, it appears that, from a medical and sociological point of view, recommending universal vaccination of women, especially girls and young women, is an appropriate health measure, and it was introduced into vaccination schedules following pharmaco-economic and opportunity analyses. However, recommending the universal vaccination of girls and young women may entail ethical problems that may make it difficult to implement these campaigns. Assessing these ethical considerations will constitute the main part of our paper.

## Discussion

### Ethical considerations with respect to the infection and its vaccine

Some of the potential ethical issues that HPV vaccination may give rise to are as follows:

#### Possible existence of negative side effects

The side effects reported after administration of the vaccine have been mainly local reactions, while signs or symptoms of greater importance have been reported in very rare cases [33,39,41]. Since the FDA and the EMEA approved the vaccine in 2006, more than 120,000,000 people worldwide have been vaccinated, and there have been no known deaths due to the vaccine preparation [42].

Furthermore, taking into account that the vaccine has an efficacy of practically 100% for preventing precancerous lesions caused by the viral genotypes included in the vaccine [32-35] and that the adverse events reported did not endanger the health of the adolescents, these side effects cannot be a sufficient contraindication for universal vaccination as the vaccine's benefits far outweigh any possible harm. Even in the case in which a woman has already had contact with one type of HPV and thinks that the benefits provided by the vaccine may not offset the adverse effects, the vaccine may protect her against the genital disease caused by other types of viruses included in the preparation and so its administration is still indicated.

#### Whether or not to vaccinate people who have opted to abstain from sex

Adolescents who have decided to maintain sexual abstinence, either due to education or personal choice would not technically be susceptible to HPV infection, as they are not exposed to at-risk relationships, so it would not be necessary to vaccinate them. However, absolute sexual abstinence is not an easy option to maintain and can vary throughout life, especially if we take into account that the age at which the vaccination is recommended is between 9 and 14 years old, an age at which it is unlikely that girls will have adopted a sexual behaviour that will remain unaltered throughout their life.

Furthermore, even if they remain true to that decision, this is unlikely to be maintained after marriage. Even though sexual intercourse takes place within the couple itself and is based on the fidelity and exclusivity of the spouses, it may be that one or both spouses has not maintained an attitude of sexual abstinence during their entire premarital life and so there may be the possibility that one of them is infected with HPV and could infect the healthy partner. In order to prevent this hypothetical transmission, it would have to be ensured that neither spouse was infected with HPV before the marriage, but this practice seems difficult to accomplish socially. Vaccination prior to marriage could be recommended, although, as has been mentioned, the effectiveness of the vaccine is highest if administered between the ages of 9 and 14 years because, at that age, a higher antibody titre is generated than at later ages [43]. It appears, therefore, that the time prior to marriage is not ideal for administering the vaccine. Consequently, not only does it appear advisable to vaccinate but it should also be done in the most effective age range.

#### Presumption of early onset of sexual relations for recommending the vaccination

The recommendation for the universal HPV vaccination of girls and young women is based, on the one hand, on the possible early onset of sexual relations, [44] and, on the other, because studies on the vaccine's effectiveness in relation to the age at vaccination have shown that the effectiveness is lower when the vaccine is given after the age of 9–14 years [38]. In both circumstances, the universal vaccination of girls and young women appears justified [32,33,45]. However, given that from any point of view it is considered advisable to delay the age of onset of sexual relations, the recommendation for universal vaccination should be accompanied by educational campaigns that recommend delaying sexual relations, and should also promote awareness of the serious medical consequences of HPV infection [46] and other risky behaviours.

Be that as it may, sometimes universal HPV vaccination may be part of a family or public strategy that seeks to facilitate the onset of sexual relations in young people, sparing them greater risks, especially the danger of developing cervical cancer. In relation to this, even if vaccination were part of that strategy, the vaccination in itself would not be unethical; it is the strategy that would be unethical, i.e. the ethical problem would have to be shifted to the strategy that forms the context of the vaccination and not the vaccination itself, because obviously the instrumentalisation (using something that is not your own for a purpose) of vaccination programmes does have an ethical content, or because it deliberately encourages a good thing to mislead and promote something bad (“safe sex”), or because it prevents a good thing so that it cannot be exploited for something bad (“safe sex”). Both choices are unethical and are not justified by the possible good intention.

#### How the interests of the commercial firms that manufacture the vaccine could affect its use

When making an ethical reflection on the advisability of a universal vaccination policy for girls and young women, the interests of the commercial firms that manufacture the vaccines, as well as the intermediary agents, and to what extent the economic benefits for both could influence decision-making should be taken fully into account. In fact, it is essential that, in order to ensure the correct choice of vaccine and the programmes to be implemented, the administration gives priority to the economic efficiency of the potential vaccines to be used – this would guarantee the suitability of the vaccination programmes chosen.

It should also be taken into account that the research processes required to develop viable vaccines are very costly. At present, the main economic investment for the development of new vaccines is made by the commercial firms and so, in order to maintain the investment, the vaccines obtained must be marketed effectively. It is obvious that if they do not achieve high use of the vaccines, they cannot continue investing in research. The information on HPV infection given to the general public should be improved, which is why many companies carry out information campaigns on HPV and its vaccine together with their vaccination campaigns [47]. Information campaigns on HPV are an effective way to improve the vaccination effectiveness. If the vaccine is not sold, users will not be able to benefit from the advances of medical research companies. For this reason, it is important to determine which vaccines are useful for the population and to use them in vaccination programmes to provide feedback to the scientific-economic process and, in this way, to be able to continue to gain improvements and medical breakthroughs in the future.

#### Advisability of proposing universal vaccination in males

The massive vaccination of males might be one measure that could reinforce the results of vaccine protection [45,48]. Although males do not have a risk of cervical cancer, they form part of the epidemiological chain of infection because they are essential elements in virus transmission between people. For this reason, collective immunity may be fundamental in the infection of women and its subsequent consequences.

Moreover, previous studies have assessed the inclusion of males in the vaccination programmes and have found a cost-benefit relationship that is sufficient to opt for inclusion, [49,50] although the setting in which these studies were conducted was, and is, changing. It is obvious that there is a different setting in each region and so new evaluations in different areas could modify any decisions taken in the future. Furthermore, there are other cancers associated with chronic HPV infection that do directly affect men, such as cancers localised in the penis, anus or oropharynx, which is why the vaccination of men must continue to be evaluated.

### Ethical considerations on the advisability of promoting universal vaccination of girls and young women

#### General aspects

When making an ethical assessment on whether to promote universal campaigns in favour of the vaccination of girls and young women with the HPV vaccine, in our opinion, the first question that arises is whether it can be considered ethical to remove the deterrent effect that is the fear of acquiring a serious and, in fact, fatal illness, as a result of the universal use of the HPV vaccine, and whether the disappearance of this deterrent effect could promote sexual relations among adolescents and young people. In relation to this, we believe that the first question that should be asked is: “Is it ethically acceptable to base the proper use of human sexuality on the fear of contracting a serious disease (consequently discouraging the use of a medical action, which in itself does not entail any direct ethical difficulty) because of the possibility that said medical action might reduce the hypothetical fear of developing the disease, which could indirectly contribute to encouraging sexual relations in young people when the deterrent effect represented by the possibility of infection and development of this disease disappears?” In other words: “Can we stop using a useful medical measure to prevent a disease – a measure that in itself does not have any ethical difficulty – amid fears that it could indirectly encourage sexual promiscuity in young people?” This is a question that, specifically in relation to the HPV vaccine, could be asked as follows: “Is it ethically acceptable not to promote universal HPV vaccination in girls and young women for fear that this could reduce the deterrent effect – i.e. the fear of developing cervical cancer – and that this could encourage sexual relations in young people?”

#### Introductory moral reflexion

Human sexuality is far from being solely a physiological mechanism for reproduction, undoubtedly an extremely important end in itself, but it is also an expression of physical and spiritual love between a man and a woman. It is not only an instrument to generate pleasure, but is also an expression of human love, hence its greatness, which goes well beyond a mere reproductive mechanism.

Due to the physical and spiritual nature of sexuality, it follows that its exercise cannot be evaluated exclusively from a biological aspect, but should also be assessed from an ethical point of view. To that end, the ethical aspect of the exercise of human sexuality forces us to consider this when evaluating actions that affect it in some way – this is true in the case of the HPV vaccine. Therefore, any action or act related to human sexuality requires an ethical evaluation.

We are of the opinion that the ethical evaluation on the use of the HPV vaccine, in principle, could be classified within the so-called “double effect” actions. “Double effect” actions are those that simultaneously cause two effects, one of which is morally positive and the other negative.

Traditionally, it has been postulated [51] that to assess the moral lawfulness of a “double effect” action, four conditions are required: a) that the action in itself be positive or at least indifferent; b) that the negative secondary effect may not be the cause or the means to achieve the positive primary effect; c) that there is no other valid alternative; d) that there be a certain proportionally grave reason between the positive and the negative effect. However, a more specific interpretation of the “double effect” moral principle has recently been proposed, reducing it to whether there is a “proportionate reason” for carrying out the direct action [52]. Thus, in the first place, we have to assess whether the specific action of universal HPV vaccination can be effectively treated using the moral figure of “double effect” actions.

In our opinion, we believe that the vaccination cannot be classified within the moral judgement merited by “double effect” actions, since the effect sought – i.e. to prevent an infection that may cause a serious or even fatal disease, something that can be achieved with the vaccination – is not accompanied by any negative side effects that could morally invalidate the positive primary effect sought. However, in certain circles, it is argued that proposing universal HPV vaccination, especially in adolescents, could encourage sexual relations between adolescents by removing the deterrent effect of the fear of contracting a serious disease, something that, in our opinion, cannot be directly inferred from the very fact of the vaccination, especially if we take into account that by proposing the vaccination at early ages, this is removed in time from the possible onset of sexual relations, and so conflating vaccination and sexual activity does not appear to be justified.

Furthermore, a recent study conducted in the United States [53] assessed the sexual activity of a group of girls who had been vaccinated against HPV, comparing it with another group with similar characteristics who had not received it. According to the authors, “HPV vaccination in the recommended ages was not associated with increased sexual activity-related outcome rates”.

Likewise, there is an added circumstance that we believe may invalidate the negative ethical judgement that vaccination might merit when considering, as already indicated, that it could promote sexual promiscuity by removing the deterrent effect for preventing sexual relations between adolescents. This circumstance is that, with the use of the HPV vaccine, development of a serious disease (cervical cancer) that is acquired sexually can be reduced or prevented, but it does not prevent the spread of other sexually transmitted diseases, among them some as serious as HIV; in other words, it does not seem that, with the potential to be able to prevent one disease with the HPV vaccination but not to eliminate the risk of spread of other sexually transmitted infections, we can continue using the argument that by removing the deterrent effect, which is reducing the risk of developing cervical cancer, by eliminating a single possibility of infection, one might think that we are going to contribute to an increase in sexual relations among adolescents, if the risk of contracting other equally serious sexually transmitted diseases through these relations remains.

In addition to this, and with respect to infection with HIV and other sexually transmitted diseases, it could be argued that together with vaccination, it could promote the use of condoms and so would achieve so-called “safe sex”, i.e. adolescents could have sexual relations without the possibility of being infected, especially with HIV. However, this seems misleading, since it is well known that using condoms does not completely prevent the possibility of being infected with the virus in a heterologous couple, i.e. with one healthy partner and the other HIV-positive, as using a condom for this purpose has a failure rate of between 5% and 10% [54-60].

#### Inalienable right of parents to decide whether or not to vaccinate their daughters

One aspect that – regardless of the advisability or not of the universal vaccination of girls and young women – must be left quite certain, is the inalienable right of parents to decide about the sex education of their children, and, therefore, the parents must be provided with all the information relative to the use of the HPV vaccine and the consequences that it could have in relation to their sexual activity. It is particularly important that parents, in the context of a universal vaccination programme for adolescents, can refuse to have their daughters vaccinated if they consider it appropriate – a right that should be made explicit in the vaccination campaign. In order to ensure this parental autonomy, even if the vaccine is promoted or recommended by the public health authorities, the final decision on its use should always remain in their hands [61-63].

## Summary

In summary, we believe that proposing a universal HPV vaccination campaign in girls and young women does not entail any particular ethical difficulties, providing that the aforementioned premises are taken into account, so we do not see any major drawbacks in proposing it.

## Competing interests

Díez-Domingo, was principal investigator in clinical trials sponsored by GlaxoSmithKlein, Merk and Sanofi Pasteur MSD. The rest of authors don't have any conflict of interests.

This article has not been funded.

## Authors’ contributions

PN-I made substantial contributions to the conception and design, acquisition of data and analysis and interpretation of data. JA made substantial contributions to the ethical debate. JD-D was involved in drafting the manuscript and revising it critically for important intellectual content and has given final approval of the version to be published. All authors read and approved the final manuscript.

## Pre-publication history

The pre-publication history for this paper can be accessed here:

http://www.biomedcentral.com/1472-6939/15/29/prepub
